# Increasing Rates of Youth and Adolescent Suicide in Canadian Women

**DOI:** 10.1177/07067437211017875

**Published:** 2021-05-17

**Authors:** Sara Zulyniak, Kathryn Wiens, Andrew G. M. Bulloch, Jeanne V. A. Williams, Aysha Lukmanji, Ashley K. Dores, Leah J. Isherwood, Scott B. Patten

**Affiliations:** 1 O’Brien Centre for the Bachelor of Health Sciences, 70401 Cumming School of Medicine, University of Calgary, Alberta, Canada; 2 Dalla Lana School of Public Health, 7938 University of Toronto, Ontario, Canada; 3 Department of Community Health Sciences, 70401 Cumming School of Medicine, University of Calgary, Alberta, Canada; 4 Mathison Centre for Mental Health Research & Education, Hotchkiss Brain Institute, 70401 Cumming School of Medicine, University of Calgary, Alberta, Canada; 5 Cuthbertson & Fischer Chair in Pediatric Mental Health, 2129 University of Calgary, Alberta, Canada

**Keywords:** child and adolescent psychiatry, suicide, self-harm, epidemiology

## Background

Suicide affects all age groups. The highest rates of suicide in Canada are reported in ages 45 to 59, and suicide is the second leading cause of death in ages 15 to 34.^1^ The last national analysis of Canadian suicide and self-harm trends reported a decrease in total suicide rate (using combined sex, age-standardized rates) from 1979 to 2012.^1^ However, this trend did not apply to both sexes equally, with female suicide rates remaining relatively consistent from 1979 to 2012 in comparison to decreasing male rates.^1^ Further, the prevalence of suicidality (ideation, plans and attempts) has increased among young adult females recently.^2^ These data indicate suicidality and self-harm are increasing in females and urge further analysis of trends in recent sex-specific suicide rates in Canada. This analysis examines trends in sex-specific suicide rates from 2000 to 2018.

## Methods

Statistics Canada mortality and mid-year population data from 2000 to 2018 were used for analysis (cited in the Data Access section). The number of deaths by “intentional self-harm” (ICD codes: X60-X84, Y87) were tabulated in 10-year age groups and stratified by sex. Combined number of deaths by intentional self-harm were created for ages 10 to 19, 20 to 29, 30 to 39, and so on, to a maximum age group of 90+. Annual suicide rates from 2000 to 2018 were then calculated as the number of deaths by suicide divided by the mid-year population within each age and sex strata. Linear regression analysis was used to calculate the annual change in suicide rate for each group over the 18-year period.

## Results

**Figure 1.**
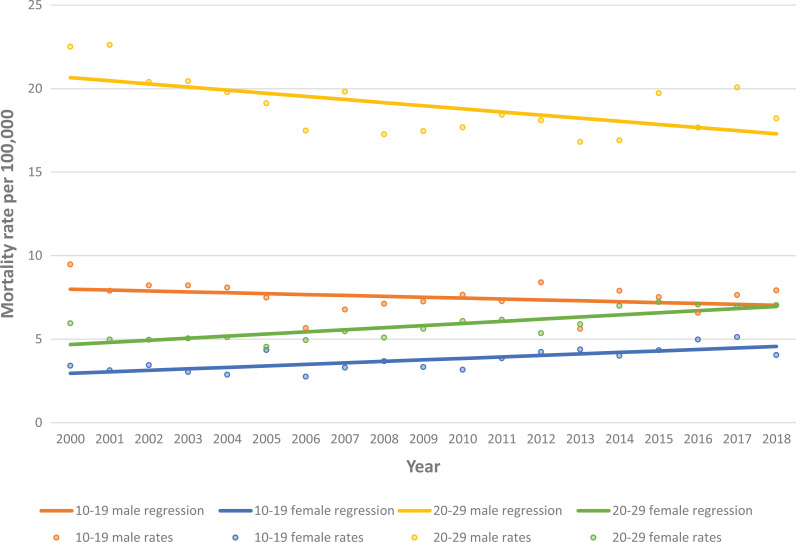
Suicide rates of individuals aged 10 to 19 and 20 to 29 from 2000 to2018 by sex.

For each year since 2000, the suicide rate has increased by 0.09 deaths per 100 000 females aged 10 to 19 (β = 0.09, *P* < 0.01) In females aged 20 to 29, every year from 2000, the rate of suicide has increased by 0.13 deaths (0.13, *P* < 0.01). In comparison, the data for males in ages 20 to 29 showed significantly decreasing rates (β = −0.19, *P* = 0.006). The 10 to 19 male group showed a non-significant decrease in suicide rate. The rates from 2000-2018 of males and females aged 10-19 and 20-29 are displayed in Figure 1. Further, the remaining age groups among males saw either decreasing (in ages 30 to 39, 40 to 49, and 70 to 79) or non-significant regression results. Additionally, no other female age groups demonstrated a significant increase in rates of suicide over the study period.

## Discussion and Conclusions

The suicide rates in females aged 10 to 19 and 20 to 29 were increasing between 2000 and 2018. In comparison, no male regression results indicated significantly increasing rates. These results indicate a worrisome trend among youth and adolescent females, supplementing past Canadian analyses that have observed steadying female suicide rates and decreasing rates of suicide among males.^1^

In order to guide public health actions, further research should attempt to classify rates in relation to methods of self-harm and suicide. Prior studies have found that suffocation is an increasingly prevalent method of suicide in both males and females, in comparison to firearm use and poisoning.^3^ Additionally, further research may aim to discern relationships between suicidality and self-harm trends in relation to mortality and sex differences, as discussed in the background. It is also currently unclear why females experience higher rates of suicidality and self-harm, though males continue to have higher rates of completed suicide.^1,2,4^

Strengths of this analysis include the use of Statistics Canada mortality data—which covers the total Canadian population. Limitations of this study include the possible misclassification or underrepresentation of suicide in vital statistics registries. ICD-10 codes were used to determine suicide mortality for this analysis, and it has been previously cited that suicides are sometimes classified as accidental deaths or deaths of “unknown causes.”^5^ Such misclassification could lead to bias in the estimation of rates, but it is unclear how this may affect the rates presented.
